# Factor XIII Supplementation Suppresses Recurrent Hemorrhages Due to Acquired Factor XIII Deficiency Secondary to Severe Acute Pancreatitis: A Case Report

**DOI:** 10.7759/cureus.109148

**Published:** 2026-05-18

**Authors:** Tatsuya Tanaka, Tomohiro Abe, Yusuke Yamada, Hidenobu Ochiai

**Affiliations:** 1 Department of Emergency and Critical Care Medicine, University of Miyazaki Hospital, Miyazaki City, JPN; 2 Department of Internal Medicine, Yamada Clinic, Akune City, JPN

**Keywords:** blood transfusion, factor 13 deficiency, hemorrhage, pancreatitis, wound healing

## Abstract

Hemorrhagic complications associated with severe acute pancreatitis significantly worsen the prognosis. Acquired factor XIII (FXIII) deficiency can lead to delayed tissue repair and associated recurrent bleeding, yet its clinical impact in the context of pancreatitis remains poorly understood. A 69-year-old woman with severe acute pancreatitis developed recurrent hemorrhagic shock due to multiple arterial bleeding episodes, including the splenic and left gastric arteries. Despite repeated transcatheter arterial embolization and massive blood transfusions, hemorrhagic events recurred during the subacute phase. Laboratory investigations revealed an acquired FXIII deficiency with FXIII activity levels of 56-59%. Repeated administration of FXIII concentrates administration suppresses the hemorrhagic complications gradually. This report suggests that FXIII concentrate administration may be a beneficial therapeutic option for managing recurrent hemorrhagic complications and stabilizing the clinical course in patients with severe acute pancreatitis and acquired FXIII deficiency.

## Introduction

Acute pancreatitis is an inflammatory condition of the pancreas triggered by factors such as pancreatic duct obstruction or alcohol consumption, and it leads to peripancreatic inflammation through the release of pancreatic enzymes [1]. Severe acute pancreatitis with excessive inflammatory progression is a life-threatening condition that causes not only acute systemic complications, such as acute respiratory distress syndrome and distributive shock, but also long-term sequelae, resulting in a high mortality rate of up to approximately 40% [2]. Hemorrhagic complications are one of the major complications of acute pancreatitis, caused by enzymatic injury to vascular walls due to pancreatic necrosis or pseudocysts, or vascular damage associated with surgical interventions, which worsens the prognosis [3]. Therefore, the management of hemorrhagic complications during the clinical course of severe acute pancreatitis remains a significant challenge.

Factor XIII (FXIII) is a plasma transglutaminase that serves as the final enzyme in the coagulation cascade, stabilizing fibrin clots through cross-linking. Furthermore, FXIII plays several roles beyond hemostasis, such as wound healing and tissue repair [4]. Acquired FXIII deficiency is a condition characterized by a decrease in FXIII levels, which can result from excessive consumption during hemorrhage or major surgery, or decreased production in conditions such as liver disease. FXIII deficiency is known to be associated with delayed wound healing and a risk of rebleeding [5].

Here, we report a case of acquired FXIII deficiency with recurrent hemorrhagic complications associated with severe acute pancreatitis, in which the patient’s condition was stabilized by repeated administration of FXIII concentrate. This report suggests the potential efficacy of FXIII in interrupting the vicious cycle of FXIII depletion, subsequent delayed wound healing, and recurrent bleeding episodes during the intense inflammatory process of severe acute pancreatitis.

## Case presentation

A 69-year-old woman was transported to our hospital with a chief complaint of epigastric discomfort followed by persistent abdominal pain for one week. Her medical history included hypertension, but no history of alcohol use disorder. On arrival, her vital signs were as follows: alert consciousness; body temperature, 36.5℃; blood pressure, 157/92 mmHg; pulse rate, 78 beats/minute; respiratory rate, 22 breaths/minute; and oxygen saturation, 97% (room air). Physical examination revealed tenderness from the epigastrium to the left flank, but no clear signs of peritoneal irritation. Laboratory tests showed leukocytosis (18,600/µL) and marked elevations in amylase (2,611 U/L) and lipase (4,290 U/L) (Table 1).

**Table 1:** Laboratory findings on admission LDL: low-density lipoprotein; HDL: high-density lipoprotein

Parameters (units)	Patient Values	Reference Ranges
White blood cell count (×10^3^/μL)	18.6	3.3–8.6
Neutrophil (%)	91.2	37–72
Hemoglobin (g/dL)	15.7	13.7–16.8
Platelet count (×10^3^/μL)	264	158–348
Urea nitrogen (mg/dL)	14.8	8–20
Creatinine (mg/dL)	0.61	0.65–1.07
Sodium (mEq/L)	141	138–145
Potassium (mEq/L)	3.1	3.6–4.8
Chloride (mEq/L)	108	101–108
Calcium (mg/dL)	9.0	8.8–10.1
Total bilirubin (mg/dL)	0.9	0.4–1.5
Aspartate aminotransferase (IU/L)	112	13–30
Alanine aminotransferase (IU/L)	65	10–42
Lactate dehydrogenase (IU/L)	317	124–222
Alkaline phosphatase (IU/L)	114	38–113
γ-glutamyl transpeptidase (IU/L)	40	9–32
Amylases (IU/L)	2611	44–132
Lipase (IU/L)	4290	10.8–42.9
C-reactive protein (mg/dL)	0.09	0–0.14
Triglyceride (mg/dL)	96	30–117
LDL-cholesterol (mg/dL)	45	65–163
HDL-cholesterol (mg/dL)	41.1	48–103
IgG4 (mg/dL)	48.7	4.5–117
Prothrombin time (sec.)	12.5	11.4–14
Prothrombin time international normalized ratio (INR)	0.97	
Activated partial thromboplastin time (sec.)	21.0	25–35
Fibrinogen (mg/dL)	321	200–400
D-Dimer (μg/mL)	3.02	0–1
Base excess (mmol/L)	0.5	-2−2

Contrast-enhanced abdominal computed tomography (CT) showed edematous changes in the pancreas, but no significant main pancreatic duct dilation or pancreatic parenchymal necrosis (Figure 1).

**Figure 1:**
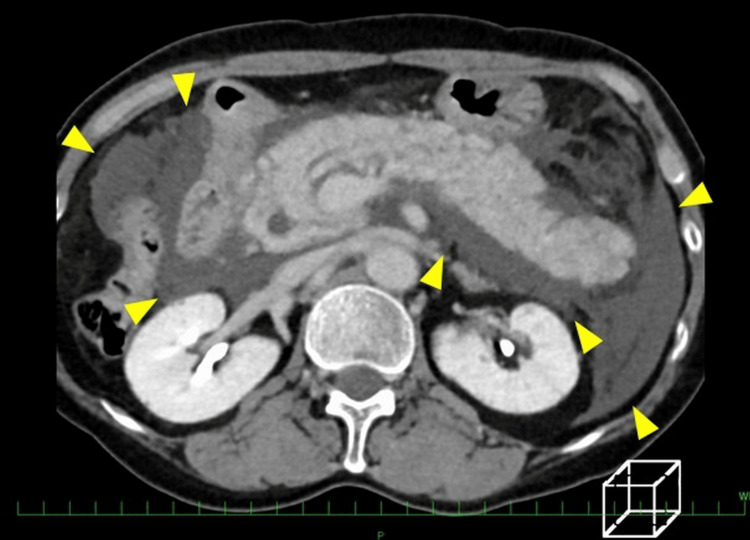
Abdominal computed tomography (CT) on arrival. Contrast-enhanced CT shows pancreatic swelling without significant main pancreatic duct dilation or areas of poor enhancement suggestive of necrotizing pancreatitis. Extensive ascites and perirenal fluid collections are evident (arrowheads).

Extensive ascites was observed around the pancreas, extending from below the diaphragm to the area below the lower pole of the kidney. Magnetic resonance cholangiopancreatography showed no findings suggestive of pancreatic duct obstruction (Figure 2). The patient had no history of alcohol use disorder, autoimmune disease, chronic abdominal pain, or the use of causative medications. Laboratory test results ruled out dyslipidemia and elevated IgG4 as causes of pancreatitis. Consequently, after excluding these other apparent etiologies, we considered the pancreatitis likely secondary to the passage of a gallstone, although total bilirubin was not elevated and alkaline phosphatase showed only a marginal increase.

**Figure 2:**
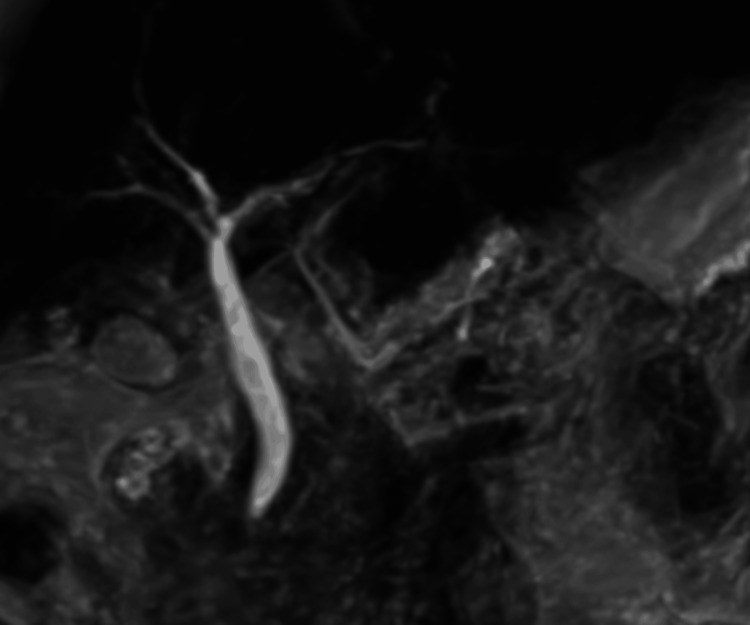
Magnetic resonance cholangiopancreatography (MRCP) MRCP reveals no significant obstructive findings, including gallstones or pancreatic duct stenosis.

Following admission, fluid resuscitation and pain management were initiated, but decreased urine output, renal dysfunction, and hyperkalemia developed by the following day. A state requiring massive fluid infusion persisted, and the intra-abdominal pressure (IAP) was measured by the transvesical method. Despite analgesia with fentanyl and gastrointestinal decompression via a nasogastric tube, the IAP reached a maximum of 31 mmHg, indicating abdominal compartment syndrome. Following endotracheal intubation, deep sedation, and the administration of a neuromuscular blocking agent, the IAP decreased to 22 mmHg.

On day 3, norepinephrine administration was started due to decreased fluid tolerance, and the patient was transitioned to deep sedation and analgesia under mechanical ventilation for intra-abdominal pressure management. On day 5, sedation was discontinued as intra-abdominal pressure decreased; however, her respiratory status acutely deteriorated that night. Diagnosed with ventilator-associated pneumonia and pleural effusion, the patient was treated with antibiotics (piperacillin/tazobactam and vancomycin) and bilateral thoracic drainage. Although a temporary improvement was observed and she was extubated on day 15, fever and respiratory failure recurred on day 20, necessitating the resumption of mechanical ventilation.

CT on day 21 showed a walled-off fluid collection around the pancreas (Figure 3).

**Figure 3:**
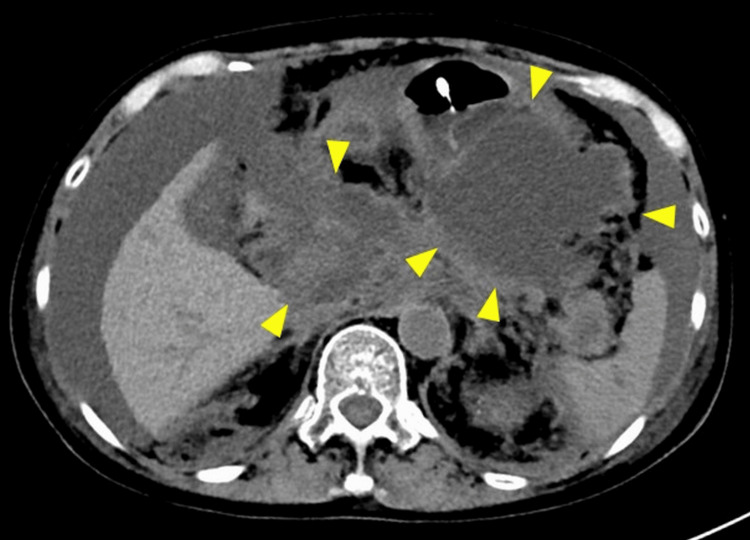
Abdominal computed tomography (CT) on day 21. CT shows a walled-off fluid collection developing in the peripancreatic area (arrowheads).

Since the collection cavity increased over time, endoscopic ultrasound-guided transluminal drainage was performed on day 41; however, drainage from the tube ceased the following day. On day 44, the fever recurred, and the patient went into shock. CT revealed a fluid collection with emphysema extending from the left retroperitoneum to the peripancreatic area (Figure 4).

**Figure 4:**
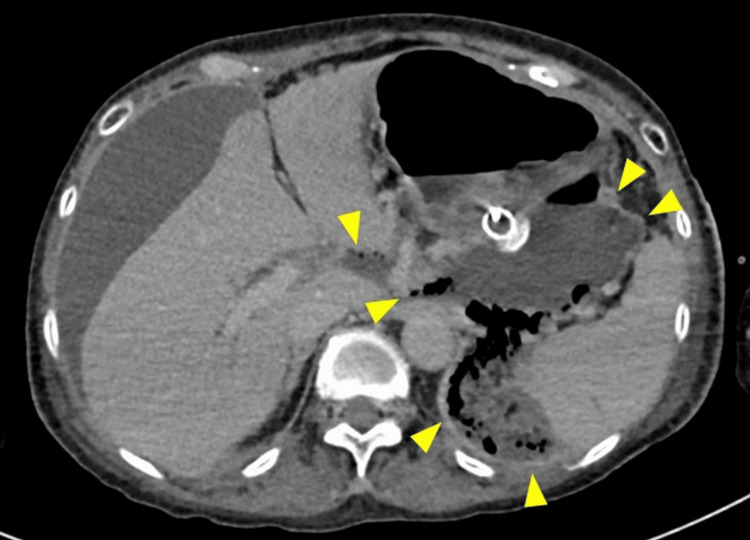
Abdominal computed tomography (CT) on day 44. CT reveals an emphysematous fluid collection extending from the left retroperitoneum to the peripancreatic area (arrowheads), suggestive of infected walled-off necrosis.

Diagnosed with septic shock due to a retroperitoneal abscess, percutaneous drainage of the left retroperitoneum was performed on the same day, and purulent infected ascites was confirmed. *Enterococcus faecalis*, *Streptococcus mitis*, and *Candida krusei* were detected in the retroperitoneal abscess, while *Staphylococcus aureus* was isolated from the pleural effusion; antibiotic therapy was continued based on sensitivity test results. On day 45, transgastric necrosectomy was attempted but proved difficult, and open drainage was performed via the left flank.

Subsequently, the patient experienced recurrent hemorrhagic complications. Bleeding from the open drainage site on days 53 and 55 led to hemorrhagic shock, necessitating a significant blood transfusion, totaling 50 units of red blood cells, 46 units of fresh frozen plasma, and 30 units of platelet concentrate, and hemostasis via transcatheter arterial embolization (TAE). On day 55, massive hematemesis occurred. An emergency laparotomy using a transgastric approach under massive transfusion revealed active arterial bleeding through the internal fistula. Angiography under resuscitative endovascular balloon occlusion of the aorta management in a hybrid operating room confirmed contrast medium extravasation from the splenic artery (Figure 5).

**Figure 5:**
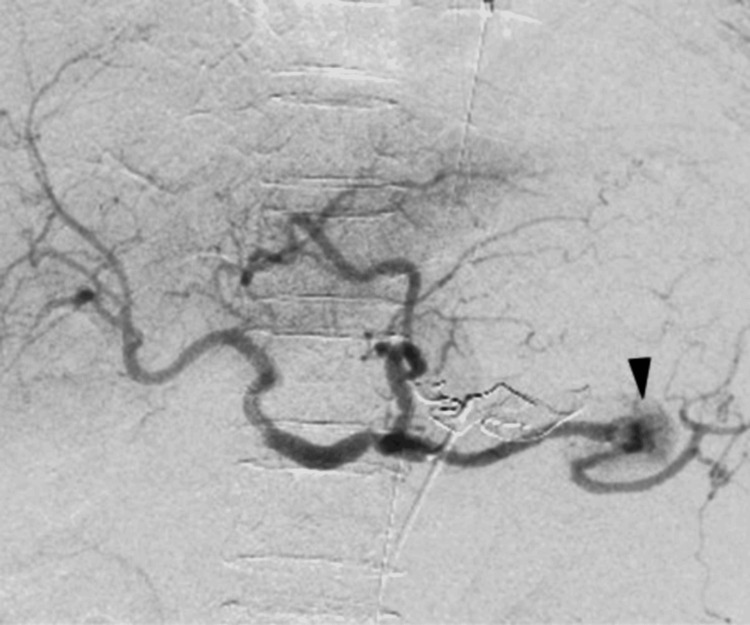
Intraoperative angiography during hemostatic surgery. Intraoperative angiography performed in a hybrid operating room showing extravasation from the splenic artery (arrowhead).

TAE was subsequently performed using coils and n-butyl cyanoacrylate. Following these events, frequent hemorrhagic episodes continued, including retroperitoneal bleeding on day 69 and bleeding from the left gastric artery on day 73 (controlled by TAE), each episode requiring further blood transfusions.

Due to these recurrent bleeding episodes, acquired FXIII deficiency was suspected, and subsequent investigation revealed a low FXIII activity of 56% (normal range: 70-140%) on day 75. The most recent fibrinogen level was 481 mg/dL, indicating no decrease. Following five days of FXIII supplementation with FXIII concentrates (fibrogammin P, 1,250 units/day) starting on day 77, the the hemorrhagic event temporarily resolved, accompanied by an increase in FXIII activity to 194%. However, bleeding from the gastroduodenal artery occurred on day 94, and from the deep circumflex iliac artery on day 119; both instances were managed with blood transfusions and arterial embolization. In both cases, while fibrinogen levels remained stable, FXIII activity was reduced (59% and 58%, respectively), necessitating further FXIII supplementation (Table 2). Each course of FXIII supplementation was temporally associated with the suppression of hemorrhagic events requiring transfusion (Figure 6).

**Table 2:** Chronological changes in coagulation profiles and factor XIII (FXIII) activity around hemorrhagic episodes. Hemorrhagic episodes occurred on Days 73, 94, and 119. Data on Days 73, 93, and 119 represent the parameters evaluated immediately prior to the onset of these hemorrhages, while data on Days 75 and 97 represent those obtained from the same blood samples collected for FXIII activity assays. PT: prothrombin time; INR: international normalised ratio

Parameters	Day 73	Day 75	Day 93	Day 97	Day 119
Platelet count (10^3^/μL)	117	176	62	34	72
PT-INR	1.49	1.16	1.45	1.31	1.37
Fibrinogen (mg/dL)	329	481	296	340	225
FXIII (%)		56		59	58

**Figure 6:**
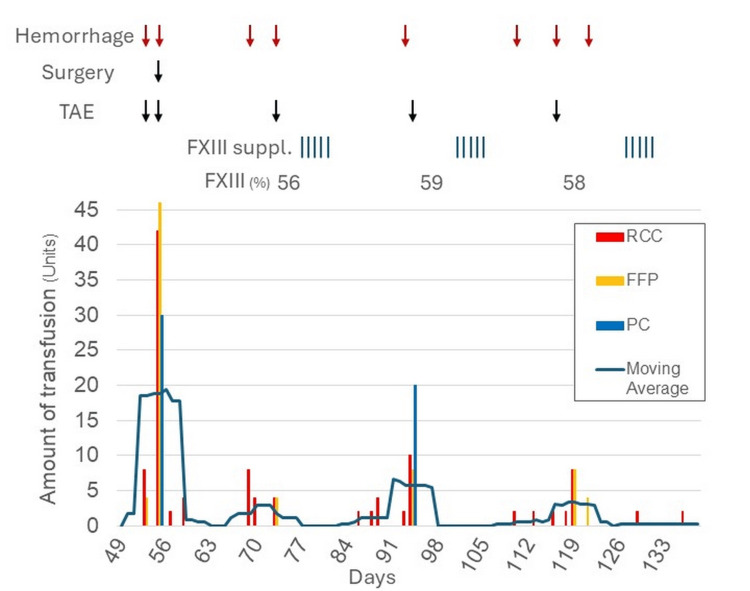
Clinical course and treatment timeline. On day 53, bleeding occurred from the retroperitoneal drainage site, requiring transfusions of RCC (red columns) and FFP (yellow columns), with hemostasis achieved via TAE. However, massive rebleeding occurred on day 55, necessitating further transfusions of RBC, FFP, and PC (blue columns); hemostasis was managed through both laparotomy and TAE. hemorrhagic episodes requiring transfusions recurred. On day 75, FXIII activity was found to be reduced to 56%. FXIII concentrate was administered for five days from day 77 to 81, resulting in a 12-day interval until the subsequent hemorrhagic event. Further bleeding events occurred on days 93, 110, 116, 119, and 121. Due to recurrent decreases in FXIII activity on days 97 and 119, FXIII concentrate was re-administered on days 101–105 and 122–126. Each administration of FXIII concentrate elongated the interval between hemorrhagic events and consequently reduced total blood transfusion requirements (represented by the solid line indicating the moving average). FPP: fresh frozen plasma; ECC: red cell concentrate; TAE: transcatheter arterial embolization; PC: platelet concentrate; FXIII: factor XIII

Despite these efforts, the patient developed progressive multi-organ failure and disuse syndrome over the long clinical course. As the treatment goals were deemed unattainable, the management strategy was transitioned to palliative care. On day 138, the patient was transferred back to the referring hospital.

## Discussion

This case represents an instance of recurrent hemorrhagic complications following severe acute pancreatitis, where the administration of FXIII concentrate appeared to extend the intervals between bleeding episodes. In acute pancreatitis, necrosis of the pancreatic parenchyma and peripancreatic tissues, combined with prolonged inflammation, delays tissue repair and results in vascular damage. These processes involve complex, intertwined mechanisms, including not only inflammation but also thrombus formation and activation of the fibrinolytic system [6]. FXIII is an essential factor in the early stages of tissue repair; however, its levels are known to decrease primarily in hemorrhagic diseases. Furthermore, FXIII depletion occurs not only in systemic inflammatory conditions like sepsis but also under localized inflammatory states such as inflammatory bowel disease [5]. This is thought to result mainly from local consumption of FXIII during inflammation-associated coagulation activation, microthrombus formation, and tissue repair processes. In our patient, systemic FXIII deficiency was confirmed during the subacute phase. This suggests that the initial high-grade systemic inflammation of acute pancreatitis, followed by local inflammation from infected walled-off necrosis, led to systemic FXIII consumption. Additionally, the concurrent hemorrhagic events likely accelerated FXIII depletion, resulting in a vicious cycle of delayed tissue repair and further bleeding. 

In our presented case, the repeated administration of FXIII concentrates approximately every two weeks was temporally associated with an extension of the intervals between hemorrhagic complications, although the levels of FXIII were relatively low. In general, low FXIII levels of less than 10% are associated with hemorrhagic complications; therefore, FXIII concentrate is primarily indicated for the prevention and treatment of hemorrhage in congenital FXIII deficiency. As in our case, FXIII supplementation is also expected to promote tissue repair in cases of delayed wound healing associated with acquired FXIII deficiency [7]. To our knowledge, no reports have specifically addressed the efficacy of FXIII supplementation in acute pancreatitis. However, a study of 50 patients with postoperative pancreatic fistula and decreased FXIII levels found that early FXIII supplementation did not effectively shorten the duration of drainage [8]. This result may have been influenced by the relatively controlled nature of surgical injuries or the study design, in which the control group also received FXIII concentrate during the chronic phase (after day 14). Severe burns represent a systemic inflammatory state comparable to severe acute pancreatitis. A randomized controlled trial of 40 patients with severe burns showed that those who received FXIII concentrate had significantly reduced perioperative transfusion requirements without any associated thrombotic events [9]. This finding supports the potential efficacy of FXIII concentrates in conditions where recurrent bleeding episodes follow intense inflammation, as seen in our case. Therefore, in cases such as ours, characterized by extreme systemic and local inflammation and recurrent hemorrhage, FXIII concentrate administration may be a beneficial therapeutic option.

This report has several limitations that must be considered. First, FXIII supplementation in acquired FXIII deficiency does not act as a primary or immediate hemostatic measure. In the event of massive hemorrhage, simultaneous interventions such as blood transfusion and surgical or radiological source control are essential, as demonstrated in this case. FXIII was supplemented primarily to support tissue repair and stabilize the fibrin network rather than to achieve acute hemostasis. Second, it cannot be definitively concluded that FXIII supplementation improved the patient’s overall systemic prognosis. The timing of the onset of acquired FXIII deficiency and its replacement may vary, and its impact on the progression of multi-organ failure remains unclear. Finally, as this is a single case report involving a complex interplay of recurrent bleeding, severe inflammation, and infection, our observations may not be generalizable to all patients with severe acute pancreatitis. Therefore, further clinical studies are warranted to evaluate the efficacy and optimal timing of FXIII supplementation in this population.

## Conclusions

Severe acute pancreatitis triggers a deleterious vicious cycle where systemic and localized inflammation depletes FXIII, thereby impairing tissue repair and exacerbating recurrent hemorrhage. In our case, FXIII supplementation was temporally associated with an extension of the intervals between recurrent bleeding episodes, suggesting its potential benefit in interrupting this vicious cycle. These observations suggest that FXIII administration may offer a strategic intervention to support tissue integrity in refractory inflammatory states, such as severe burns. Further studies are warranted to evaluate the clinical utility of therapeutic FXIII supplementation for acquired FXIII deficiency in severe inflammatory diseases.
